# A *Candida albicans *early stage biofilm detachment event in rich medium

**DOI:** 10.1186/1471-2180-9-25

**Published:** 2009-02-02

**Authors:** Adnane Sellam, Thamir Al-Niemi, Kathleen McInnerney, Susan Brumfield, Andre Nantel, Peter A Suci

**Affiliations:** 1 Biotechnology Research Institute, National Research Council of Canada, Montreal, Quebec, Canada; 2 Department of Plant Sciences and Plant Pathology, Montana State University, Bozeman, MT 59717, USA; 3 Genomics Core Facility, Department of Microbiology, Montana State University, Bozeman, MT 59717, USA; 4 Department of Microbiology and Center for Biofilm Engineering, Montana State University, Bozeman, MT 59717, USA

## Abstract

**Background:**

Dispersal from *Candida albicans *biofilms that colonize catheters is implicated as a primary factor in the link between contaminated catheters and life threatening blood stream infections (BSI). Appropriate in vitro *C. albicans *biofilm models are needed to probe factors that induce detachment events.

**Results:**

Using a flow through system to culture *C. albicans *biofilms we characterized a detachment process which culminates in dissociation of an entire early stage biofilm from a silicone elastomer surface. We analyzed the transcriptome response at time points that bracketed an abrupt transition in which a strong adhesive association with the surface is weakened in the initial stages of the process, and also compared batch and biofilm cultures at relevant time points. K means analysis of the time course array data revealed categories of genes with similar patterns of expression that were associated with adhesion, biofilm formation and glycoprotein biosynthesis. Compared to batch cultures the biofilm showed a pattern of expression of metabolic genes that was similar to the *C. albicans *response to hypoxia. However, the loss of strong adhesion was not obviously influenced by either the availability of oxygen in the medium or at the silicone elastomer surface. The detachment phenotype of mutant strains in which selected genes were either deleted or overexpressed was characterized. The microarray data indicated that changes associated with the detachment process were complex and, consistent with this assessment, we were unable to demonstrate that transcriptional regulation of any single gene was essential for loss of the strong adhesive association.

**Conclusion:**

The massive dispersal of the early stage biofilm from a biomaterial surface that we observed is not orchestrated at the level of transcriptional regulation in an obvious manner, or is only regulated at this level by a small subpopulation of cells that mediate adhesion to the surface.

## Background

Members of the *Candida *genus are the principal etiological agents of nosocomial fungal infections, with *C. albicans *being the most common species [1-3]. The overall mortality rate for patients with candidemia is greater than 40% [4-6]. Catheters are considered to be a likely point of entry of *C. albicans *into the vascular system [7]. In support of this evaluation, a particularly high risk of invasive candidiasis is associated with the use of urinary and vascular catheters, and ventricular assist devices [8]. The chances of acquiring a BSI resulting from colonization of an intravascular catheter by Candida species has been ranked high among pathogens involved in biomaterial centered infections, second only to *Staphylococcus aureus *[9]. *C. albicans *colonizes various biomaterials and readily forms dense, complex biofilms under a variety of in vitro conditions [10]. *C. albicans *biofilms exhibiting similar architectural and morphological features form in vivo [11-13]. The implication is that dissemination from *C. albicans *biofilms colonizing biomaterials is frequently a major factor predisposing susceptible patients to life threatening BSI.

Despite the evidence that dispersal of cells from *C. albicans *biofilms may be a critical step in biomaterial related cases of candidemia, few studies have characterized *C. albicans *biofilm detachment behavior. Daughter cells that are released from *C. albicans *biofilms cultured on cellulose acetate filters or cellulose fibers perfused with a continuous flow of medium have been collected either as a means to assess biofilm growth rate [14], or to determine if dispersed cells retain the intrinsic (transient) phenotypic resistance to antimicrobials that is a hallmark of biofilms [15]. In the former study there is an implicit (untested) hypothesis that the detachment rate is constrained by the medium substrate loading rate, and not simply a direct (passive) response to the applied (mechanical) shear force. Expression of a GPI (glycosylphosphaditylinositol) anchored cell wall protein (Ywp1p) has been shown to decrease adhesion between biofilms composed of yeast forms and a polystyrene surface [16]. The implication is that Ywp1p may be the effective structural component in an active control network that induces biofilm detachment. A recent review has discussed cell dispersal from *C. albicans *biofilms with respect to its possible induction by farnesol, a quorum sensing agent that promotes formation of the yeast form [17]. *C. albicans *biofilms formed from mutants in which genes coding for key adhesins under the positive control of the Bcr1p transcription factor have been disrupted produce thin fragile biofilms [11,18]. Detachment of cells from biofilms formed from these mutant strains is significantly enhanced [19].

Evidence is accumulating that bacterial biofilms actively regulate dispersion processes using a variety of mechanisms [20-28]. The aim of the present study was to determine if we could find evidence indicating that *C. albicans *biofilm detachment from a biomaterial surface was actively regulated at the level of transcription. A clearly observable, reproducible transition between establishment of strong adhesion and loss of adhesion in a relatively copious early stage biofilm provided us with a simple tractable in vitro system for probing changes in the transcriptome associated with loss of adhesive bonds to a biomaterial. Since the phenomenon involved the entire biofilm population we could apply a relatively simple scheme for array analysis which consisted of a closed loop time course comparison. A comparison of biofilm and batch cultures provided us with an additional way to screen for genes that were specifically involved in the detachment process.

## Results

### The detachment process involves an early abrupt loss of strong adhesion

**Figure 1:**
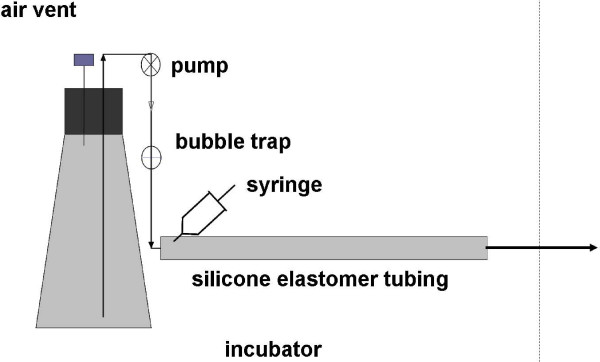
**Biofilm tubular reactor**. The reactor was inoculated by drawing a cell suspension into the tube from the effluent end (arrow) using a sterile syringe inserted through the tubing wall just down stream from the bubble trap. The bubble trap also serves as a sterility barrier. The entire system was enclosed in an incubator for temperature control (broken line).

**Figure 2:**
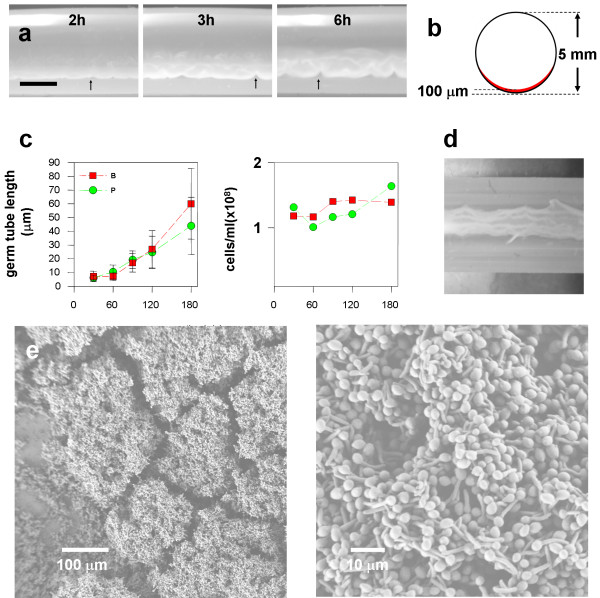
**Biofilm detachment process**. a) Images of the biofilm in the tubing (side view) at various time points; regions where the biofilm has been displaced from the lower surface are indicated by arrows; a similar pattern of detachment was observed in more than 100 experiments; the scale bar is 2 mm; b) Approximate dimensions of the 3 h biofilm with respect to the tubing cross section; c) Changes in germ tube and cell density versus time; Legend: B, biofilm, P, planktonic (batch) culture; (By 1 h 82% and 76% of the cells geminated in biofilm and planktonic cultures, respectively);d) The multilayer biofilm that remains on the surface after draining at times up until 1 h extends along the trough of the tubing and is visible; (Representative digital camera image of the underside of the tubing); e) SEM images of the 1 h biofilm that remains on the lower surface of the tubing after draining.

Biofilms were cultured in a tubular reactor similar to that used in a previous study [29] (Figure 1). Figure 2a shows stages of biofilm detachment that are evident from visual inspection of the silicone elastomer tubing in which the biofilms were cultured. Regions where the biofilm has been displaced from the tubing become visible by 2 h and continue to enlarge during the course of development. These regions of detachment are evident along the entire length of the tubing. Biofilms cultured for 6 h appear to have only minimal points of contact with the silicone elastomer. Typically, this tenuous association is completely lost between 8 and 9 h, at which point the entire biofilm is displaced downstream by the flow.

As in other *C. albicans *biofilm studies [11,30-33], our inoculum was produced at 30°C in order to obtain a well defined dispersed population consisting entirely of yeast singlets and doublets, with no cell aggregates. This relatively large inoculum settles to the lower surface of the tubing during the 1 h incubation period. These cells, which still have the yeast morphology after the 1 h incubation period, are completely removed if the tubing is drained, leaving the lower tubing surface completely free of cells (data not shown). Contrary to our initial expectation, when medium flow is initiated, most cells remain associated with the surface. We found less than 10^5 ^cells/ml in aliquots collected immediately after initiation of flow until just before loss of the entire biofilm (five experiments). Cells that remain associated with the surface germinate and the biomass increases primarily by hyphal extension rather than increase in cell number (Figure 2c). (A batch culture in which the conditions of the inoculation are the same behaves similarly in this respect). Biofilms grown for 1 h have developed a multilayer, multicellular structure that remains associated with the tubing after it is subjected to the large shear forces exerted at the interface by draining the tubing (Figs 2d and 2e), indicating that as cells germinate they rapidly develop relatively strong cell to cell (cohesive) and cell to surface (adhesive) bonds.

**Figure 3:**
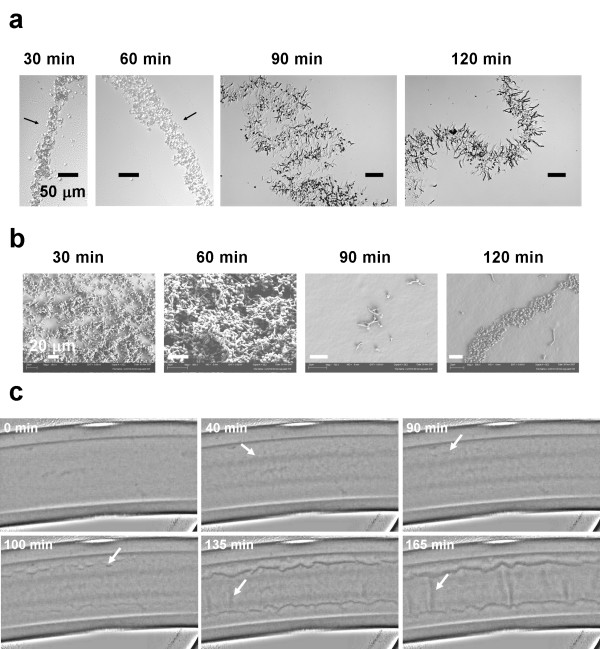
**Time course of loss of adhesion and accompanying microscopic and macroscopic structural changes**. a) Cryosections of biofilms at different time points. Sections acquired at 30 and 60 min appear to conform to the curved surface of the tubing. Arrows indicate substratum side. The structure in which hyphae at the edges extend into the surrounding medium becomes apparent between 60 and 90 min. (Scale bars are all 50 μm). b) SEM images of the colonized (lower) surface of the tubing after the tubing was drained. Between 60 and 90 min there is a sharp transition in which most of the cells have lost their surface adhesion. (Scale bars are all 20 μm). c) Time course of gross structural changes during loss of adhesion. The biofilm is visible at 40 min. At 90 min the flanking sections detach as flaps (arrow); these flaps are more visible at later time points. At 135 min wrinkles begin to form (arrow) and become more prominent at later time points (185 min).

The relatively strong adhesive association with the surface that is established by 1 h is weakened considerably before visible regions of the biofilm lift off the tubing and this is accompanied by a change in biofilm morphology. The early time course of this loss of adhesion was followed using cryosectioning, scanning electron microscopy (SEM) and time lapse photography (Figure 3). Cryosections of the biofilm indicated that there was a fairly abrupt transition in the structural organization of regions of the biofilm (particularly regions near the biofilm lateral edges) consisting of the appearance of hyphae extending into the surrounding medium between 60 and 90 min (Figure 3a). Characterization of the surface using SEM showed that the abrupt structural transition was coincident with the loss of adhesion to the surface (Figure 3b). At 30 and 60 min a multilayer biofilm remained after draining the tubing while at later time points (90 and 120 min) most of the cells were displaced by draining. No cells could be found on the lower (previously colonized) surface after draining tubing containing a 3 h biofilm (data not shown). Time lapse photography of the top of the biofilm during the transition indicated that macroscopic detachment was first visible at the edges of the biofilm as wavy flaps (Figure 3c). At later times wrinkles appeared in the biofilm that, when viewed from the side, were evidently locations at which portions of the biofilm had been entirely displaced from the surface.

**Figure 4:**
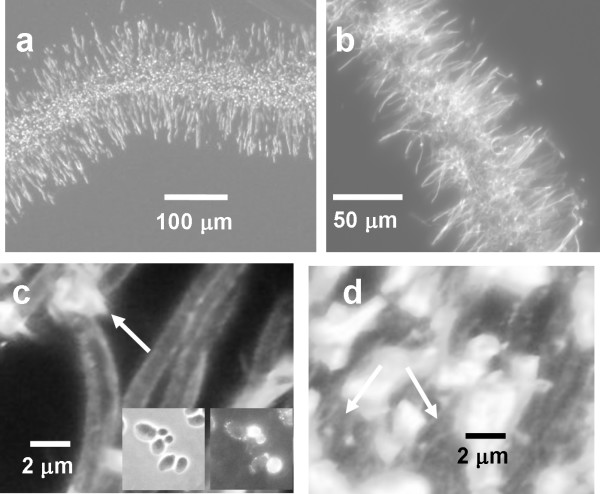
**Detached biofilm structure (3 h biofilms)**. All images were acquired using epi-fluorescence microscopy. a) Spurr's embedded section; the fluorescence originates from the preparation technique; b) Cryosection stained with calcofluor white; c, d) Cryosections stained for (1,3) β glucan; c) Region at the edge of the biofilm; arrow indicates extracellular material that is stained; inset is the planktonic positive control (left: transmitted image right: epi-fluorescence image); d) region in the interior of the biofilm; arrows indicate stained material that appears as strands.

The structural reorganization observed at the 90 and 120 min time points becomes more pronounced as the biofilm develops. Sections of 3 h biofilms were obtained transverse to the direction of flow (in the plane of the tubing cross-section) (Figure 4). The structure of the sections prepared using the Spurr's embedding method (Figure 4a) appeared quite similar to those prepared using cryosectioning, a histological technique that was designed to preserve the hydrated structure (Figure 4b). Both sectioning techniques indicated a structure in which hyphae extended from both sides of the detached biofilm into the surrounding medium. Despite their relative immaturity, the 3 h biofilms showed evidence of production of extracellular polymeric substance (EPS) as indicated by staining with a monoclonal antibody against (1,3) β glucan (Figure 4c and 4d). A previous study indicated that (1,3) β glucan is a primary component of *C. albicans *EPS [34]

Table 1 summarizes the hydrodynamic (shear) forces associated with displacement of the biofilm from the tubing at various stages of growth. (The approximate dimensions of the 3 h biofilm with respect to the tubing were indicated in Figure 2b). The yeast inoculum was not rinsed from the surface by the relatively low shear force (0.016 dyn cm^-1^) of the medium flow which is an indication that this hydrodynamic force is quite gentle. However, it was completely displaced from the surface by draining the tubing (data not shown). In contrast biofilms cultured for between 30 min and 1 h have established a sufficiently firm adhesion so that the biofilm can withstand application of a substantial shear force (17.3 dynes/cm^2^).

**Table 1:** Hydrodynamics of biofilm displacement from the surface

	Shear Force (dynes/cm^2^)^1^	
	0.016	17.3
Yeast inoculum^2^	+	-
30 min-1 h Biofilm	+	+
2–6 h Biofilm	+	-
> 8 h Biofilm	-	-

^1^Computed as indicated in the Methods section

^2 ^At the end of the 1 h inoculation period

+ biofilm remains attached

- biofilm is removed

### Initial biofilm adhesion is dependent on expression of BCR1 and ALS3 but not on HWP1

**Figure 5:**
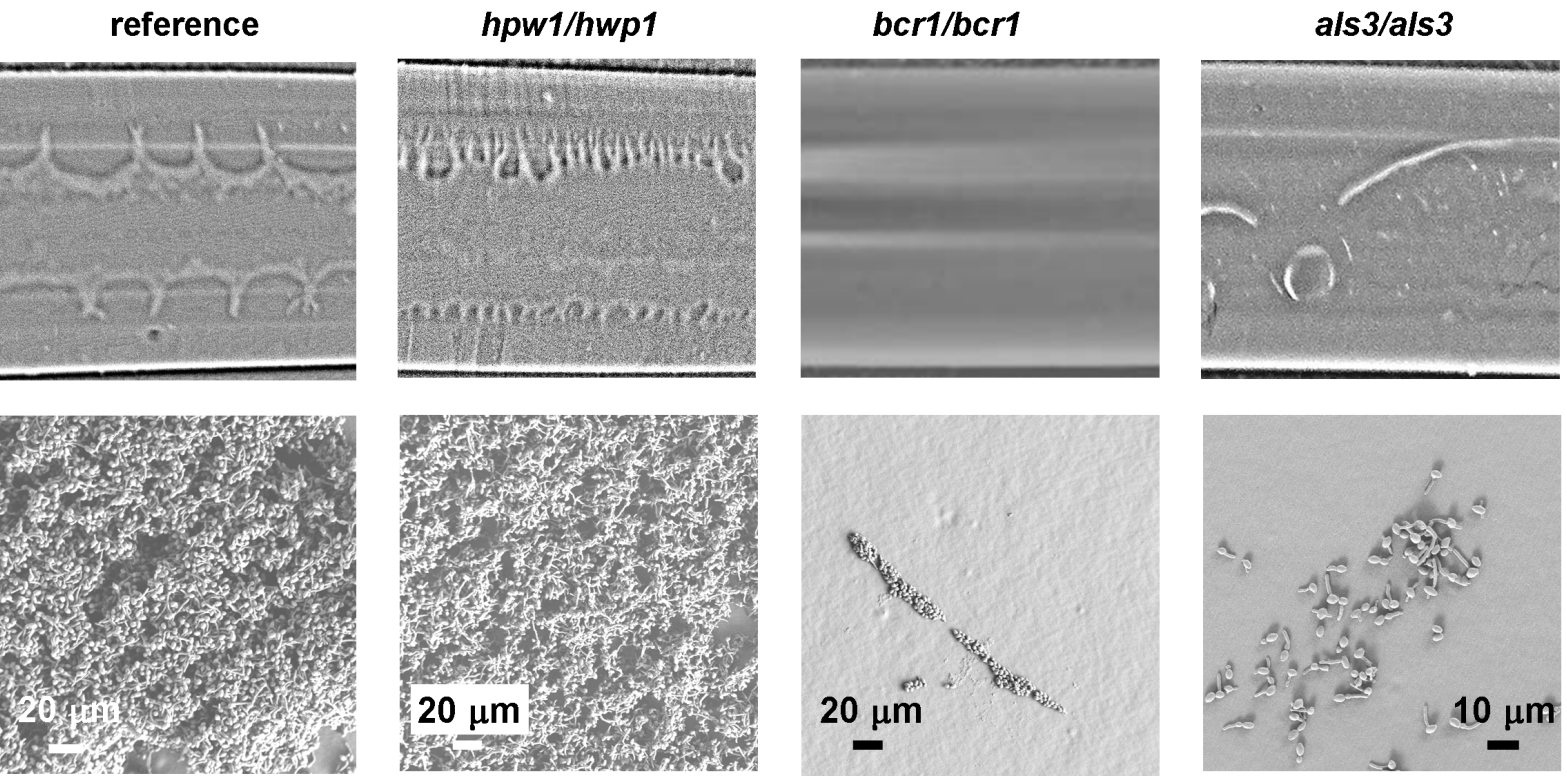
**Influence of deletion of *HWP1*, *BCR1 *and *ALS3 *and on establishment of early firm adhesion**. Biofilms formed from the strains indicated at the top of each column were harvested at 40 min and the tubing was drained. The top row are pictures of the underside of the tubing. Visible biofilm remained after draining the tubing for the reference strain (DAY286) and the *hwp1/hwp1 *mutant, while no visible biofilm remained for the *bcr1/bcr1 *mutant. There was some residual biofilm left after draining the tubing colonized by the *als3/als3 *mutant (before the ethanol rinse steps), but the adhesion to the surface was clearly much less than the reference strain. SEM images of the tubing in the second row indicated that multilayer biofilm remained on the surface of the tubing for the reference strain and the *hpw1/hpw1 *mutant, while very few cells could be found for the *bcr1/bcr1 *and *als3/als3 *mutants. The most heavily colonized regions that were found are shown. (The ethanol dehydration removed all visible biofilm from the tubing for *bcr1/bcr1 *and *als3/als3 *mutant strains).

A simple hypothesis is that the loss of adhesion described above involves a temporal shift in expression of two adhesins (*ALS3 *and *HWP1*), regulated by the *BCR1 *transcription factor, that were shown play a prominent role in *C. albicans *biofilm development [11,19,35]. In order to pursue this idea we first determined if these genes were involved in establishment of the initial strong adhesive bond to the surface. Figure 5 shows that at 40 min the reference (wild type) strain has established adhesion to the tubing surface while the *bcr1/bcr1 *and *als3/als3 *mutant biofilms are almost completely displaced from the surface by draining the tubing. *BCR1 *is a positive regulator of morphogenesis. However, the lack of establishment of adhesion of *bcr1/bcr1 *and *als3/als3 *strains was not entirely coupled to filamentation in a simple manner since a substantial proportion of the *bcr1/bcr1 *and *als3/als3 *mutant cells germinated (20 and 70%) during the 40 min time interval. (The mean germ tube length of these cells was 14 +/- 12 and 10 +/- 7 μm, respectively). The results for the *hwp1/hwp1 *mutant indicated that expression of this gene was not essential for establishment of firm adhesion, i.e., under our conditions and at this early stage in biofilm development. At 40 min the biofilm was multilayered and clearly attached. These results led us to characterize the detachment phenotype of a strain that overexpressed *ALS3 *which is described below.

### Comparison of the firmly and loosely attached biofilm suggests that glycosylation, vesicle trafficking and transport contribute to the adhesive phenotype

As shown in Figure (2d and 2e) a visible multilayered biofilm structure withstands the substantial shear force applied by draining the tubing for biofilms cultured for 1 h. A portion of the 1 h biofilms is typically removed from the surface by this procedure. These two subpopulations are referred to as the 1 h firmly (1h F) and 1 h loosely (1h L) attached biofilm. We reasoned that comparing the transcriptional profiles of these two subpopulations might uncover genes that were subsequently differentially regulated to mediate detachment in our flow model. The comparison of 1h F and 1h L biofilms revealed 22 upregulated and 3 repressed transcripts (see Additional file 1). Upregulated genes fell into process ontological categories of vesicular trafficking, glycosylation and transport. RT-qPCR confirmed the changes in transcript levels of some genes enriched in glycosylation and vesicle trafficking functions that exhibited relatively small fold changes (Table 2). The distinct pattern of expression of these genes within the context of the time course analysis is discussed in the next section.

**Table 2:** Genes up regulated in the 1hF/1hL comparison

Gene	Orf	**Microarray**^1^	**RT Q-PCR**^2^
***Vesicular trafficking***			
*SSS1*	orf19.6828.1	1.56	1.63 ± 0.01
*ERV29*	orf19.4579	1.60	3.73 ± 0.41
*SEC22*	orf19.479.2	1.44	2.24 ± 0.1
*EMP24*	orf19.6293	1.44	1.24 ± 0.1
*CHS7*	orf19.2444	1.44	1.65 ± 0.12
*YOP1*	orf19.2168.3	1.55	1.67 ± 0.15
			
***Glycosylation***			
*PMT4*	orf19.4109	1.63	ND^3^
*DPM2*	orf19.1203.1	1.61	2.33 ± 0.11
*DPM3*	orf19.4600.1	1.48	2.12 ± 0.2
*WBP1*	orf19.2298	1.44	4.75 ± 0.11
			
***Transport***			
*ADP1*	orf19.459	1.68	ND
*CTR1*	orf19.3646	1.54	ND
*ADY2*	orf19.1224	1.69	ND
*TNA1*	orf19.2397	1.68	ND
*ALP1*	orf19.2337	1.58	ND

^1^Average fold change

^2^Log_2 _ratios. Each value is the mean ± standard deviation of two independent experiments each with three replicates.

^3^Not Done

### Time course analysis indicates detachment is associated with coordinated expression of mutually exclusive functional categories

**Figure 6:**
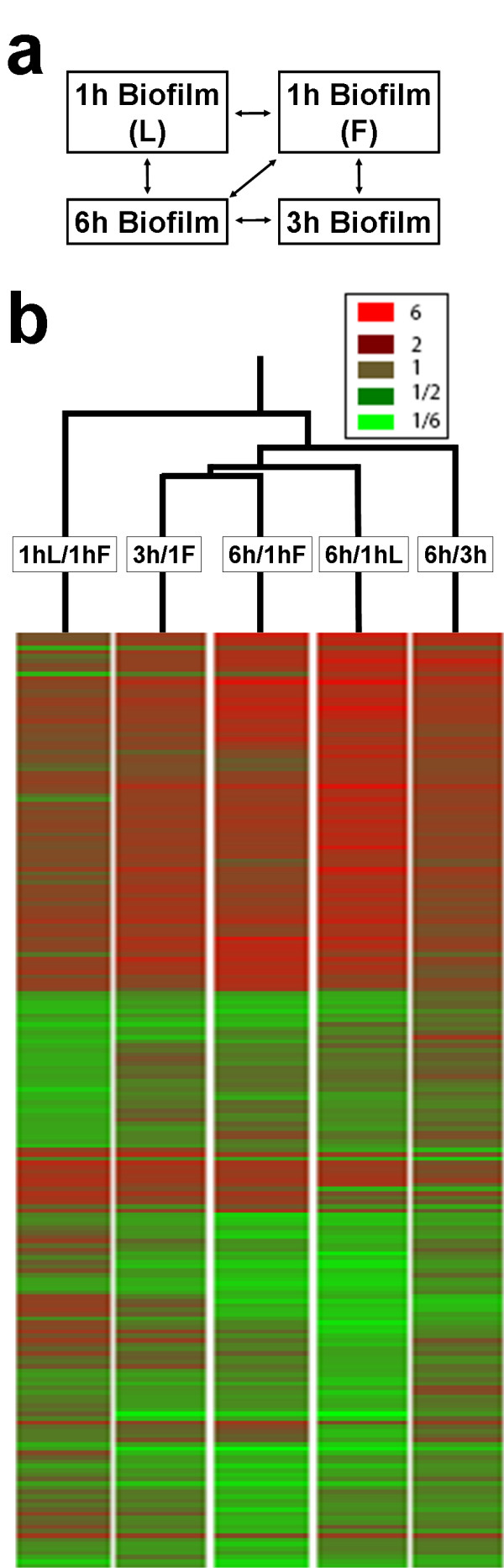
**Time course analysis on DNA microarrays**. A) Closed loop scheme. B) Heat map and two-dimensional hierarchical clustering of the different transcriptional profiles. Upregulated and downregulated genes are colored in red or green respectively.

In order to identify changes in the transcriptome that accompanied the abrupt detachment event we performed a closed loop time course analysis (Figure 6a). In such a comparison, each sample is compared to two or more other conditions thus allowing us to visually validate the changes in transcript abundance. We compared the transcriptome of 1h F and 1h L biofilms with biofilms that had spontaneously and progressively lost their adhesive bonds (3 and 6 h). The time course array analysis produced 148 predicted ORFs that were differentially regulated (>= 1.5 fold change, P-value < 0.05) for at least one pair wise comparison (Figure 6b). (The complete list of genes that are significantly modulated in each comparison is presented in Additional file 1). Of the 148 differentially regulated genes, 98 have a known inferred function. There were also 34 genes that were significantly up or down regulated in more than one pair wise condition (see Additional file 1). Comparison with two previous studies [36,37] in which cells were transferred from 30°C to 37°C in YPD medium indicated that differentially regulated genes in the time course were not associated with this temperature shift.

**Figure 7:**
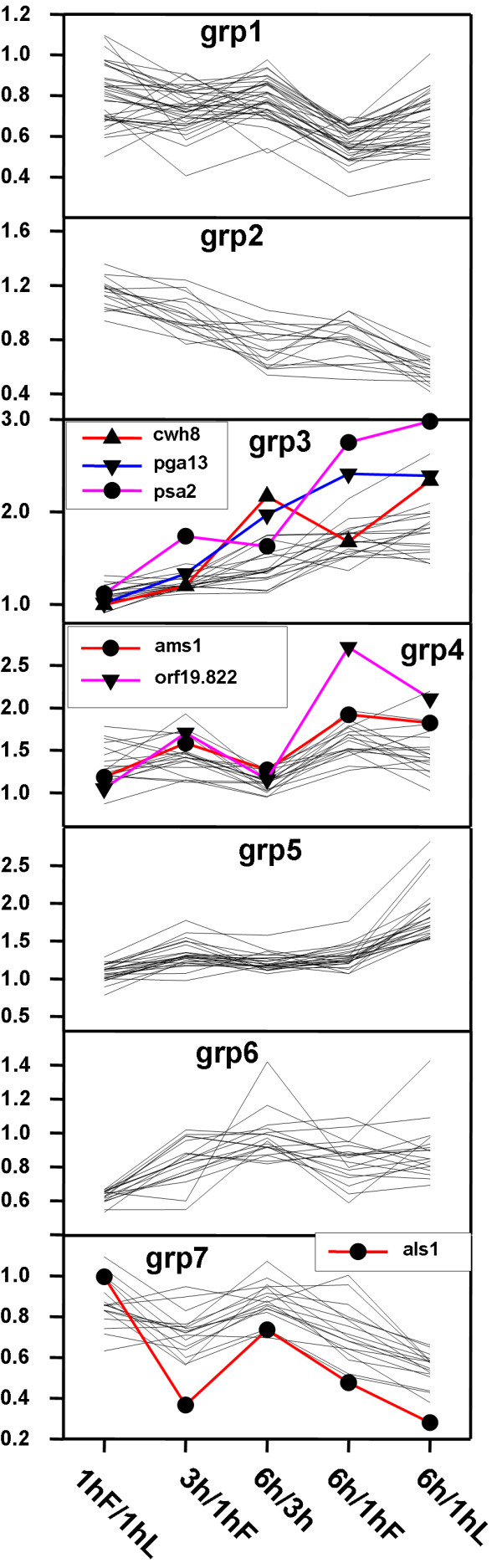
**Categories of genes with similar expression patterns identified by K means analysis**. The seven groups of genes fall into distinct ontological process categories summarized in Table 3. Patterns of expression of genes chosen for further analysis (groups 3, 4 and 7) are indicated.

K means analysis produced the most meaningful patterns in the time course array data (Figure 7). Since expression levels of all 148 genes for all conditions were included in this analysis, an implicit assumption in the interpretation is that differences in gene expression levels detected between 6 and 1 h and 6 and 3 h are a temporal extension of the differential expression pattern exhibited between 3 and 1 h. The hierarchical cluster analysis presented in Figure 6 provides some support for this assumption since it indicates that differences in expression levels between 1 to 3 h and 1 and 6 h are relatively closely related. The outlying location of the 1hL/1hF condition can be interpreted as indicating that differential transcript expression between these two groups should be treated as a separate category. In support of this interpretation we were unable to correlate genes differentially regulated during the time course analysis to genes identified in the comparison of the 1 h firmly (1h F) and 1 h loosely (1h L) attached biofilms. The proximity of the 6 h/1hF and 6 h/1hL conditions indicates it is valid to regard these two categories as reflecting similar temporal trends in differential expression.

The seven groups of genes identified by K means analysis fell into distinct GO process categories summarized in Table 3, which shows the four or five most significant categories according to the P value. With a few exceptions, the GO process category assignments for each group were mutually exclusive which suggests that the patterns uncovered by the K means analysis were functionally meaningful. Categories related to carbohydrate biosynthetic processes (group 3) and interaction with the host, adhesion during symbiosis and adhesion to the host (group 7) have the most obvious possible functional relevance to the detachment phenomenon.

**Table 3:** Ontological categories associated with groups of genes identified by K means analysis of the time course array data

Process GO term	**Enrichment**^1^	*P *value
Group 1 (17/37)^2^		
*Chromatin assembly/disassembly*	18.07	7.41e-5
*DNA packaging*	10.13	0.00011
*DNA metabolic process*	4.69	0.00114
*Regulation of meiosis*	39.0	0.00155
		
Group 2 (12/17)		
*Response to stimulus*	4.85	0.00063
*Regulation of biological quality*	8.76	0.00087
*Pseudohyphal growth*	20.75	0.00487
*Response to stress*	4.82	0.00727
*Cell growth*	15.09	0.00783
		
Group 3 (13/22)		
*Carbohydrate biosynthetic process*	12.75	0.01118
*Glycoprotein biosynthetic process*	9.00	0.02203
*Glycoprotein metabolic process*	8.50	0.02260
*Response to simulus*	2.98	0.03761
*Response to stress*	3.33	0.05641
*Cellular carbohydrate metabolic process*	4.25	0.08011
		
Group 4 (12/20)		
*Heme metabolic process*	55.33	0.00066
*Heme biosynthetic process*	55.33	0.00066
*Tetrapyrrole biosynthetic process*	55.33	0.00087
*Porphyrin biosynthetic process*	41.50	0.00087
*Porphyrin metabolic process*	41.50	0.00112
*Tetrapyrrole metabolic process*	41.50	0.00112
		
Group 5 (10/24)		
*Energy derivation/oxidation of organic compounds*	11.1111	0.00216
*Generation of precursor metabolites*	8.5714	0.00459
*Aspartate family amino acid metabolism*	18.1818	0.00519
*Sulfur metabolic process*	16.6667	0.00661
*Alcohol metabolic process*	6.8966	0.03450
*Metabolic process*	1.4706	0.05460
		
Group 6 (9/18)		
*Aerobic respiration*	19.5882	0.00041
*Cellular respiration*	19.5882	0.00043
*Energy derivation/oxidation of organics*	12.3333	0.001'54
*Generation of precursor metabolites*	6.3429	0.00330
*Pathogenesis*	6.3429	0.03922
*Interspecies interaction*	4.9333	0.06136
		
Group 7 (12/18)		
*Interaction with host*	17.5263	5.91e-5
*Adhesion during symbiosis*	31.2500	0.00014
*Adhesion to host*	31.2500	0.00014
*Biological adhesion*	20.8333	0.00039
*Pathogenesis*	9.5143	0.00065
*Single species biofilm formation/biomaterial*	41.5000	0.00139

^1 ^Ratio of the abundance in the differentially regulated gene set to the abundance in the set of genes contained in the gene ontology data base

^2 ^Total number of classified genes/total number of genes in the group

### Comparison with batch cultures indicates differential regulation of genes associated with a response to hypoxia, amino acid biosynthesis and cell surface proteins

**Figure 8:**
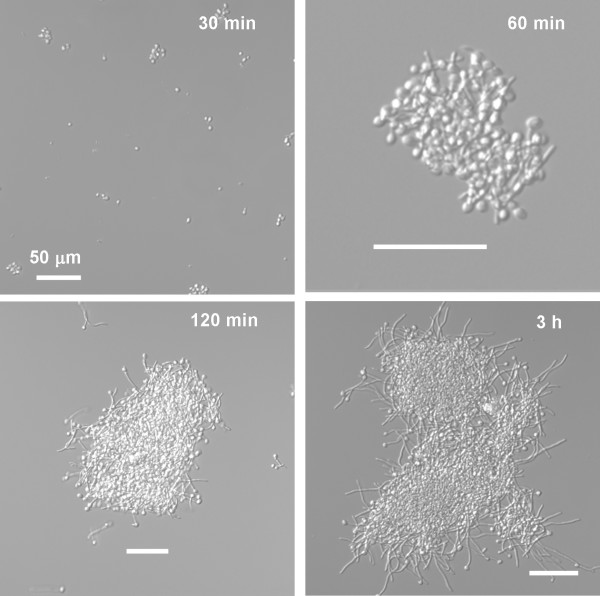
**Cell aggregate formation in batch cultures; we did not observe alignment of germ tubes extending into the surrounding medium at the edge of any of the cell aggregates**.

If inoculation conditions are made as identical as possible, batch cultures are similar to the biofilm in terms of both cell morphology (Figure 2c) and aggregation behavior (Figure 8). Since cells in the batch cultures germinate and also exhibit cohesive (cell to cell) interactions we reasoned that genes differentially regulated in the biofilm to batch comparison and the time course analysis might contain a subset of genes involved more specifically in the detachment process, rather than exclusively in morphogenesis or cell to cell cohesion. It is conventional to compare biofilm and planktonic cultures in microarray analyses, where the planktonic culture(s) serves as a sort of reference [30,33,38]. We compared 1 h and 3 h biofilm and batch cultures to each other since these time points bracketed the abrupt transition in which strong adhesion was lost. We used the 1h F biofilm for this comparison since we were attempting to uncover genes involved in mediating adhesive interactions.

**Figure 9:**
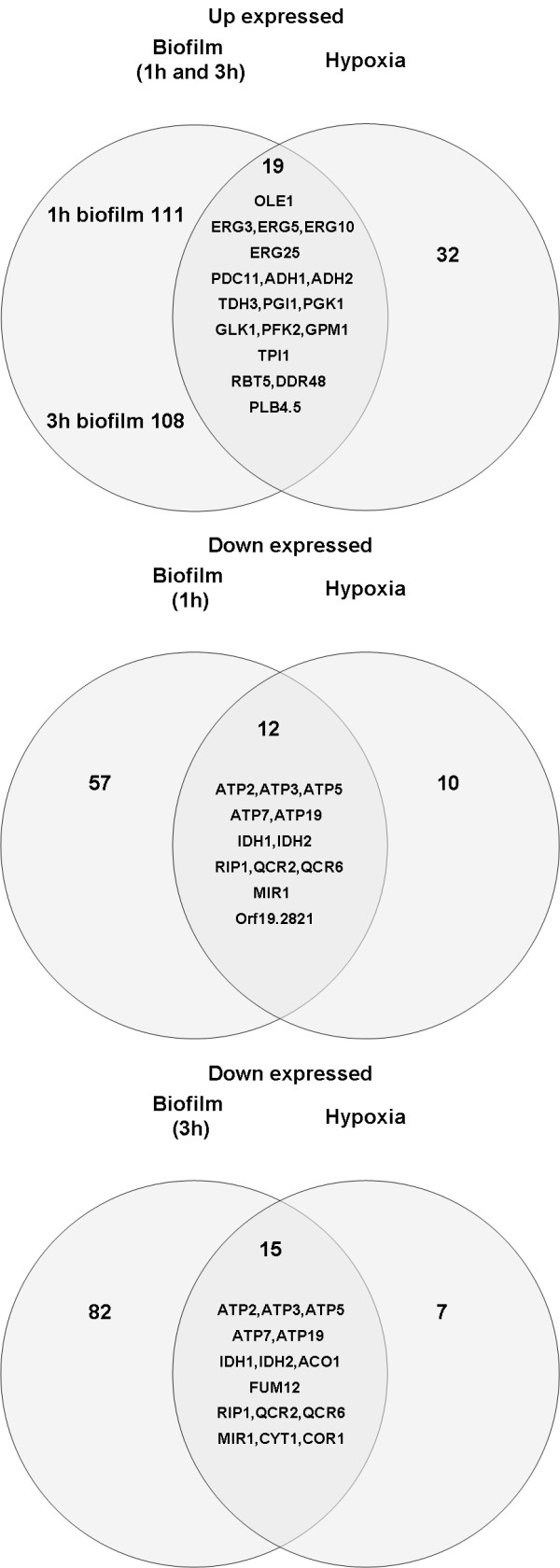
**Common differentially regulated genes in 1 h and 3 h biofilm to batch comparison and *C. albicans *cells growing under hypoxic condition**.

The categories of genes that were differentially regulated between the biofilm and batch cultures are summarized in Table 4. (The complete list of differentially regulated genes is given in Additional file 2). In general, genes coding for proteins involved in glycolysis, fermentation and ergosterol synthesis were upregulated while genes associated with oxidative phosphorylation and the TCA cycle were downregulated. This pattern of differential gene expression is very similar to that observed in comparisons of batch cultures grown under aerobic and relatively anaerobic conditions [39] and indicates that biofilm cells were responding to hypoxia (Figure 9). The batch comparison data were ordered with respect to the ratio of the fold changes at the 3 h and 1 h time points. There were 16 genes for which this ratio was greater than 1.5 or less than 0.66 and also appeared in the list of significantly regulated genes in the time course analysis. The 11 genes for which the ratio (3 h/1 h) was greater than 1.5 exhibited a pattern of expression that was fairly tightly clustered, similar to the group 4 pattern found by K means analysis (data not shown). Among these 11 genes were four which coded for proteins involved in response to stress: *ASR1*, *CDR4*, orf19.822 and *AMS1*.

**Table 4:** Summary of differentially regulated genes in the biofilm-batch comparison

Process GO Term	Genes on microarray dataset		**Annotated Genes**^1^	*P *value	
	1h-biofilm	3h-biofilm		1h-biofilm	3h-biofilm
Up regulated genes	130	127			
***Lipid metabolism***	**21**	**18**			
*Ergosterol biosynthesis*	11	9	28	1.82 E-10	6.67 E-08
*Fatty acid metabolism*	3	4	74	0.2	0.1
*Other lipid metabolism*	7	5	-	-	-
***Glycolysis***	**13**	**7**	**16**	5.74 E-18	1.75 E-07
***Fermentation***	**3**	**2**	**16**	0.01	0.07
***Amino acid biosynthesis***	**11**	**5**	**205**		
*Glutamate*	5	1	13	2.37 E-05	0.27
*Leucine*	2	0	5	8.21 E-03	-
*Other*	4	4	-	-	-
***Transport***	**12**	**4**			
*Glucose transport*	5	0	21	3 E-04	-
*Oligopeptide transport*	3	0	11	3 E-03	-
Other	4	4	-	-	-
***Cell wall***	**8**	**8**	**92**	4.5 E-03	7.7 E-03
***Protein refolding***	**0**	**5**	**14**	**-**	**9 E-05**
Down regulated genes	71	95			
***Translation (cytosolic ribosome)***	**11**	**33**	**105**		
*Large subunit*	8	18	50	9.08 E-05	8.1 E-15
*Small subunit*	3	15	39	0.08	4.68 E-13
***Tricarboxylic acid cycle***	**6**	**2**	**20**	**1.75 E-05**	**0.11**
***Amino acid biosynthesis***	**3**	**13**			
*Glutamate*	0	4	13	-	6.2 E-04
*Leucine*	0	2	5		9 E-03
*Other*	3	7	-	-	-
***ATP synthesis***	**6**	**9**	**20**	**1.75 E-05**	**4.9 E-09**
***Respiratory chain***	**8**	**11**	**26**	**5.36 E-07**	**2.02 E-10**
***Stress response***	**4**	**5**	**-**	**-**	**-**

^1^Number of genes in the annotated database

### Loss of strong adhesion is not influenced by oxygen availability at the interface or in the medium

The porous structure of silicone elastomers results in a high gas permeability [40]. (Silicone elastomer is 25 times as permeable to oxygen as natural rubber). Thus it is likely that oxygen penetration at the tubing surface might establish a gradient of oxygen at the biofilm/surface interface. The timing of the structural transition in which hyphae extending from the edges of the biofilm were first observed corresponds with the loss of adhesion (Figure 3) suggesting that the two phenomena might be related.

We tested the hypothesis that availability of oxygen at the biofilm/surface interface was providing a stimulus to induce detachment by placing a gas tight glass sleeve around the biofilm reactor and filling the sleeve with nitrogen gas. Nitrogen was induced after 40 min of growth to allow time for the biofilm to establish firm adhesion to the surface. The presence of the nitrogen had a measurable effect on hyphal length which was reduced by 62% compared to the standard conditions (29 μm versus 47 μm, p value 1.4 e-6). However, there was no visible difference in the detachment phenotype at 3 h. We performed additional experiments to see if we could perturb the detachment phenotype by availability of oxygen by either filling the glass sleeve with pure oxygen or saturating the medium with pure oxygen during biofilm development. Although there were subtle perturbations in the biofilm structure (data not shown) the detachment phenotype was not appreciably altered.

### Mutant strain analysis suggests that transcriptional regulation of a single gene candidate is not responsible for mediating the loss of strong adhesion

Based on the array analysis presented above we chose seven genes (*AMS1*, *PSA2*, *CWH8*, *PGA13*, orf19.822, *AQY1*, and *ALS1*) for further analysis. (A *cwh8/cwh8 *mutant could not be produced since it formed a trisomic suggesting that it is a lethal mutation). In addition to genes indicated by our array analysis, we chose two genes for further study based on their possible function in the detachment process as suggested by previous work (*YWP1 *and *MKC1*) [16,41]. We also constructed a strain expressing *ALS3 *constitutively under the control of the *ACT1 *promoter based on its role in establishment of the initial firm adhesion of 1 h biofilms (*ALS3 *also exhibited down expression in the time course analysis that was just below the 1.5 fold change threshold).

**Figure 10:**
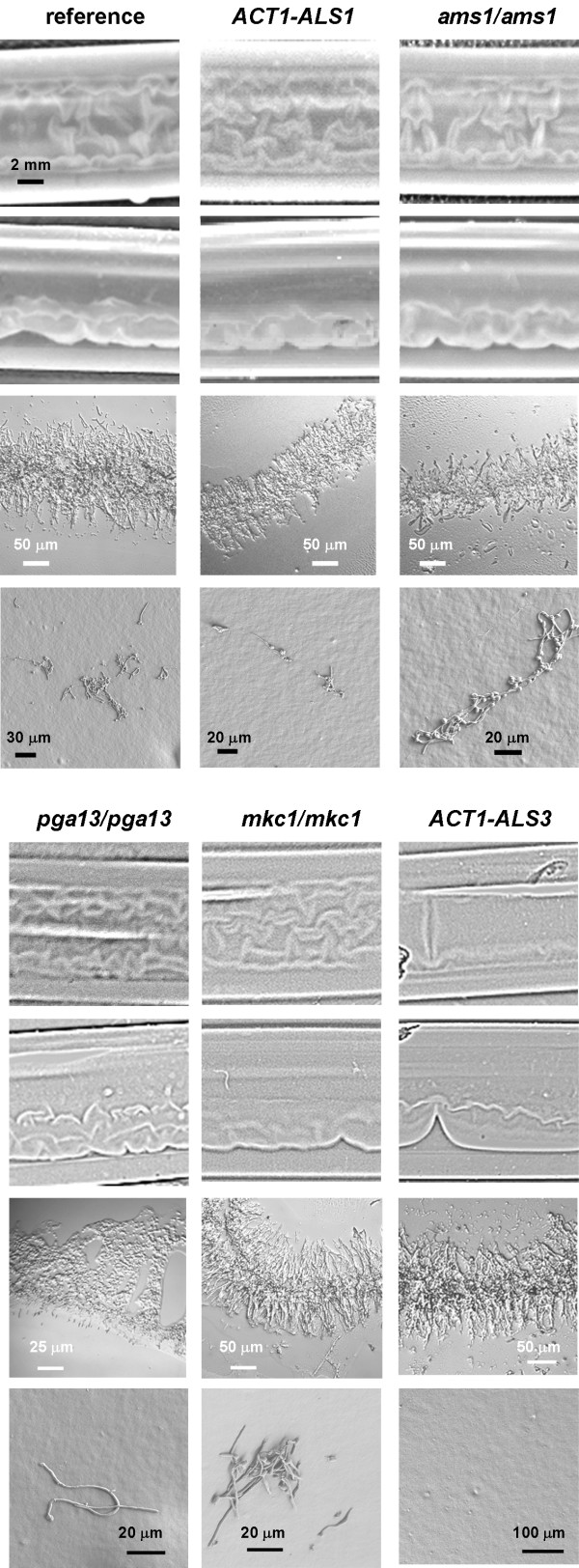
**Detachment phenotypes of selected mutants**. All data presented are for 3 h biofilms. The top row of panels in each set are digital camera images (top view, first row; side view, second row). The third row in each set are cryosections and the forth row are SEM images of the surface after draining the tubing. SEM images show the most densely colonized regions of the surface that could be found. The biofilm formed from the *pga13/pga13 *mutant was relatively fragile and this may have contributed to the altered structure of the cryosections. In terms of gross structure the most pronounced differences were seen in the *ACT1-ALS3 *construct which exhibited fewer regions of detachment that were relatively more displaced from the surface.

The detachment phenotype of nine mutant strains was characterized using visual inspection (recorded with a digital camera), cryosections of 3 h biofilms, and SEM of the surface after draining the tubing. With slight variations, all the mutant strains exhibited detachment phenotypes that were quite similar. Figure 10 presents a panel of results for six of the strains tested. In the top row are mutants exhibiting detachment phenotypes that we consider essentially identical. The detachment phenotypes of the *aqy1/aqy1 *and *ywp1/ywp1*mutants and the orf19.822 double knockout were very similar to those shown in the top row. The macroscopic appearance of the *psa2/psa2 *mutant was similar to the reference strain but the biofilm was too fragile to withstand the application of the OCT polymer to the surface so cryosections could not be obtained. In the bottom row are detachment phenotypes that exhibited slight variations. Cryosections of the *pga13/pga13 *mutant did not produce hyphae that were clearly aligned at both edges of the biofilm. We tentatively attribute this to disruption of the structure during application of the OCT polymer since this biofilm had the appearance of being more fragile than that of the reference strain. In contrast, the *mkc1/mkc1 *mutant produced a biofilm in which alignment of hyphae appeared to be more pronounced than in the reference strain. (The detachment phenotype of the CAI4 reference strain was the same as the BWP1 reference strain). The detachment phenotype of *ACT1-ALS3 *biofilm was the only one that differed appreciably from the reference strain in terms of macroscopic appearance. Compared to the reference strain this mutant exhibited fewer regions of detachment that were relatively more displaced from the surface.

## Discussion

Although circumstantial evidence strongly implicates that detachment from *C. albicans *biofilms plays a significant role in biomaterials centered infections, there is virtually nothing known about which types of detachment events might play a role, which conditions induce detachment, or the characteristics of the biofilms responsible for the dissemination. Our biofilm model is most relevant to detachment events that might occur from vascular catheters which commonly transport a relatively rich nutrient broth (total parenteral nutrition) and are statistically among the most likely prosthetic devices to be associated with *C. albicans *BSI [8]. A comparison with previous results suggests that at this early stage the biofilm is at a critical stage where it can either loose its adhesive association with the silicone tubing or develop into a mature biofilm [29]. In order to have a tractable in vitro biofilm model we used an inoculum density that is higher than that expected under any conceivable hospital conditions. However, it is quite plausible that microcolonies that develop from a much smaller inoculum might respond similarly to a constant supply of rich medium and undergo a similar process of global detachment very early in their development. It is also reasonable to expect that the primary colonizers would have previously experienced a lower temperature environment such as the skin or a hospital room.

From a medical point of view, we would like to know the interplay of factors (extrinsic and intrinsic) that trigger different types of detachment events. The perception of biofilms as structured [10] differentiated [17] communities that may exhibit developmental stages that are actively programmed [42] suggests that explicit intrinsic (regulatory) components might play a role. Two time course studies have provided a foundation for discovering points of active regulation of *C. albicans *biofilm developmental processes at the transcriptional level. Significant changes in the transcriptome accompany both the establishment of initial association with the surface [33] and precede the stage of pronounced increase in biomass [38]. This study is the first to address transcriptome changes that accompany a clearly observable biofilm detachment process. We have found that a transition in which a firm attachment to the surface is abruptly lost are coincident with changes in the transcriptome, and we have identified genes that are reasonable candidates for playing a role in this detachment. Furthermore, a subset of the genes that were differentially regulated during the transition is not associated with either hyphal extension, the most obvious morphological change at the cellular level, or cell aggregation. The microarray data indicated that changes associated with the detachment process were complex and, even after using the array data as a guide for mutant strain construction, we were unable to demonstrate that transcriptional regulation of any single gene was essential for loss of strong adhesion.

The most direct evidence that biofilm developmental processes are actively controlled by biofilm-specific transcriptional regulatory networks has come from studies of *BCR1 *dependent genes [11]. Bcr1p, a zinc finger domain transcription factor under the positive control of Tec1p, a positive regulator of morphogenesis, activates a set of genes (*ALS1*, *ALS3 *and *HWP1*) that code for GPI anchored cell surface proteins that are required for normal biofilm formation. Of these, *ALS3 *and *HWP1 *appear to play the most prominent role in biofilm development [11,19,35], and evidence suggests that their differential expression could play a role in mediating detachment events [19]. We found that *BCR1 *was necessary for establishment of adhesion of the *C. albicans *biofilm to the silicone elastomer surface and that *ALS3 *was necessary for establishment of firm adhesion, while *HWP1 *was not required. Although there was a slight trend of decreased expression of *TEC1 *in the time course analysis, there was no indication that *BCR1 *was differentially regulated during detachment and overexpression of *ALS3 *had only a modest effect on the detachment phenotype.

The time course analysis indicated that the detachment process coincided with differential regulation of a relative abundance of genes coding for plasma membrane proteins, cell surface proteins and cell wall proteins, with a modest enrichment in these categories (data not shown). These genes were scrutinized more closely for clues that would indicate changes in cell surface properties related to detachment. Genes involved in transport (*ALP1*, *TNA1*, *CTR1*, *GNP1*, *HGT1*, *HGT15*, and *DUR7*) were highly represented indicating a shift in metabolism. There was no clear trend indicating that these transcripts were generally either increased or decreased during the time course. There was a general decrease in transcripts for genes involved in hyphal penetration (*RAC1*, *PLB1*) which is suggestive of a response to reject surface association. It would be reasonable to expect that induction of release from the surface would involve cell wall restructuring and two genes (*SCW11*, *XYL2*) are related to this function.

Patterns of gene expression uncovered by K means analysis indicated that genes involved in similar biological processes were regulated together which provides some support for the hypothesis that the detachment process was associated with some form of coordinated transcriptional regulation. Genes involved in DNA packaging (*HTB1*, *HTA1*, *HHF22*, *HHT2*, and *NHP6a*) and host interaction (*RAS1*, *SAP5*, *ALS1*, and *TEC1*) were generally down regulated (groups 1 and 7, respectively), while genes involved in carbohydrate/glycoprotein (*CWH8*, *PSA2*, and *TPS3*) biosynthetic processes and energy derivation/generation of precursor metabolites (*TPS2*, *MRF1*, and *ADH5*) were generally up regulated (groups 3 and 5, respectively). Among group 1 there were a number of genes coding for histones that were found to be differentially regulated by the quorum sensing agents farnesol or tyrosol (*HTA1*, *HHT2*, and *NHP6a*), both of which have been shown to influence biofilm development [43,44].

There are a substantial number of genes whose expression levels have been shown previously to influence *C. albicans *biofilm formation. The *BCR1 *dependent genes were discussed above. The one gene (*YWP1*) specifically linked to *C. albicans *biofilm detachment [16] was notably absent from the list of differential regulated genes in the time course analysis. This was not entirely unexpected since *YWP1 *is expressed primarily in the yeast form. Another gene that was notably absent from the list was *EAP1*. The *EAP1 *gene has been shown to be required for strong adhesion to polystyrene, which is similar to silicone elastomer in that it is relatively hydrophobic [45]. *PRP22*, a gene found to be upregulated upon binding of hyphae to polystyrene [46], showed a trend of downregulation in our time course study. *PRP22 *is an RNA dependent ATP-ase, and thus probably involved in general metabolism so we did not consider this as a candidate for functional analysis.

A reasonable hypothesis is that detachment from a silicone elastomer surface is induced by a change in cell surface hydrophobicity (CSH). *C. albicans *has a variety of options for binding to host cells via specific interactions, while CSH provides a less specific means of binding to both host tissues and biomaterial surfaces [47]. Presumably cell to cell cohesion within a biofilm could be maintained by a subset of the more specific interactions, while loss of CSH would weaken adhesion to the hydrophobic silicone elastomer surface. Genes implicated in determining CSH include *CSH1 *[48,49], *MNN4 *[50] and three genes that contain an eight cysteine domain that shows similarity to a class of fungal hydrophobins (*CSA1*, *PGA10 *and *RBT5*) [32]. *CSH1 *was upregulated during the time course of detachment, a result that is difficult to interpret since this would presumably enhance binding to the silicone elastomer surface. Neither *MNN4 *nor *CSA1 *(*WAP1*) were among the genes differentially regulated in either the time course analysis or the batch comparison. *PGA10 *(*RBT51*), coding for a (putative) mannosylated GPI anchored protein, was upregulated during the time course and *RBT5*, coding for a GPI-anchored cell wall protein, was upregulated by factors of, respectively, 4.7 and 16.5 in the 1 and 3 h biofilm/batch culture comparisons, but did not appear as a significantly regulated gene in the time course analysis. (*RBT5 *was also one of the genes up regulated in response to hypoxia (5.5 fold change) in a previous study [39]).

We attempted to exploit the comparison between 1h F and 1h L biofilm subpopulations to identify additional genes that were involved in mediating adhesion with the idea that the pattern of expression of these genes during the time course might suggest genes involved in the detachment process. However, genes identified in this comparison were generally not ones that appeared in the time course analysis and, in fact, the genes in this comparison exhibited a pattern of expression that was relatively removed from the time point comparisons. This is shown both by the hierarchical clustering across the different comparisons (Figure 6), and principle components analysis (data not shown). Our interpretation is that genes identified in the comparison of 1h F and 1h L biofilm subpopulations, many of which fall into process categories of glycosylation, vesicle trafficking and transport, are candidates for mediating cohesive (cell to cell) interactions rather than adhesive (cell to surface) interactions.

The batch cultures that we used for our biofilm/batch comparison were similar to biofilms in both morphogenesis and cell aggregation behavior, and thus we anticipated that this comparison might reveal genes associated with the biofilm-specific process of detachment. A substantial proportion of the 201 genes that were differentially regulated in the biofilm/batch comparison were probably associated with a response to a relative state of hypoxia in the biofilm (Figure 9) [39]. However, expression of 12 genes coding for cell surface proteins were not differentially regulated in the previous analysis of the *C. albicans *response to hypoxia (*ALS3, CDC19, FRE10, HEM13, HSP104, HYR1*, orf19.822, *PGA10, PGA52, PGA7, PHR1*, and *SOD5*). Of these 12 genes, the most highly upregulated gene in the 3 h biofilm to batch comparison was orf19.822, a gene coding for a soluble protein that is more abundant in *C. albicans *biofilms formed on silicone elastomer than in corresponding batch cultures [51]. A notable proportion (5 of 12) of the cell surface genes code for GPI anchored or putative GPI anchored cell wall proteins (*PGA10, PGA52, PGA7 (CRW3, RBT6), PGA10 (RBT51, RBT8)*, and *HYR1*).

The patterns of gene expression across the time course conditions uncovered by K means analysis, supplemented by the biofilm/batch comparison and the inferred function suggested to us that *AMS1*, *PSA2*, *CWH8*, *PGA13*, orf19.822, *AQY1*, and *ALS1 *were candidates for playing a major role in the detachment process. Inferred functions of *AMS1*, *PSA2*, *CWH8 *and *PGA13 *indicated that these genes might play a role in restructuring the cell wall, thus possibly modifying the adhesive properties [52-54]. *AMS1 *and orf19.822 were among the genes identified as unique to the biofilm process according to the batch comparison. Orf19.822 codes for a protein that may contribute to biofilm formation on silicone elastomer surfaces [51]. We speculated that Aqy1p [55] might be one component in a system enabling an orientational response to oxygen gradients, since hyphal orientation is regulated by calcium ion channels [56] and aquaporins are proposed to have a role in cell tropism by acting in concert with ion channels to regulate cell volume changes [57]. *AQY1 *was highly up regulated in the biofilm/batch comparison. *ALS1 *was the major overexpressed gene in a detailed microarray studies that compared biofilms and batch cultures grown under a variety of conditions [30]. *ALS1 *has been described as a down stream effector of morphogenesis [58-60]. Down regulation of *ALS1 *was associated with detachment so a strain expressing *ALS1 *constitutively under the control of the *ACT1 *promoter was constructed. We also chose *ALS3 *for mutant analysis based on its apparent contribution to the establishment of firm adhesion of 1 h biofilms. In addition, we characterized the detachment behavior of mutants lacking expression of *MKC1*, a mitogen activated kinase, shown previously to be involved in surface sensing [41] and *YWP1*, a gene shown previously to be involved in detachment of yeast forms [16]. While the mutant strains *pga13/pga13*, *mkc1/mkc1 *and *ACT1-ALS3 *exhibited slight modifications in the detached biofilm phenotype, there was no strong indication that any of the gene products encoded by our candidate gene list was a primary determinant in mediating detachment.

It is possible that the detachment process we observed is not regulated at the level of transcription. Alternatively, the process could well be orchestrated by transcriptional regulation of a set of genes in a complex manner as is evident from the various interacting factors that have been shown to influence CSH [50,61-64]. An intriguing possibility is that the hyphae that extend from the edge of the detached biofilm might be phenotypically distinct from the hyphae in the interior and that this phenotypic difference is conferred at the level of transcriptional regulation. There are also numerous possible points of post-transcriptional control [65]. The first step in testing this latter hypothesis would be to compare the transcriptome with the proteome, with a focus on cell wall proteins. The fairly abrupt, clearly discernable detachment process we have described would provide an ideal system for exploring these alternative, post-transcriptional mechanisms.

The detachment processes in bacterial biofilms that show evidence of active regulation can be classified into those which are elicited by an external stimulus [23-25,66] and seeding dispersal, which occurs without applying an obvious external stimulus [27,67,68]. In this respect the detachment process we have described is similar to seeding dispersal since there is no obvious change in nutrient loading (or hydrodynamic shear stress). Evidence has been obtained that seeding dispersal is initiated by a change in an internal microenvironment in the biofilm [67]. The batch comparison indicated that biofilm cells were experiencing a relative state of hypoxia, and there was some evidence that this response was amplified during the time course of detachment. However, we found no evidence that oxygen availability was a factor in the detachment process. One possibility is that the detachment phenomenon originates from a change in hyphal cell surface properties that is a generic part of germination under these conditions. The early stage biofilm we examined did not exhibit the classic structure in which yeast are somehow sequestered at the base of the biofilm. It may be that these yeast are necessary for mediating the permanent adhesion to the surface, while hyphae provide an initial tenacious, but more transient anchor.

## Conclusion

An early stage *C. albicans *biofilm inoculated from the yeast form onto a silicone elastomer surface and grown in rich medium under flow establishes both cohesive (cell-to-cell) and relatively strong adhesive (cell-to-surface) bonds. As cells germinate and hyphae grow by linear extension the adhesive bonds are progressively weakened over an 8 h period. This loss of adhesion is accompanied by a structural reorganization of hyphae along the perimeter of the biofilm such that they become aligned in a direction perpendicular to the interfaces delineated by the biofilm-medium and biofilm-substratum boundaries. The most pronounced transition in both adhesion and structural reorganization occurs within the first 2 h of biofilm development.

A K means analysis of microarray time course data indicated that changes in the transcriptome that accompany the loss of adhesion fell into mutually exclusive functional categories. The most relevant categories were judged to be adhesion, biofilm formation and glycoprotein biosynthesis. There was no obvious pattern to suggest that a single gene regulated the detachment process. Consistent with this finding, a functional analysis using mutant strains did not reveal any striking changes in the detachment phenotype upon deletion or overexpression of key genes.

At this point in our understanding of *C. albicans *biofilm detachment it is uncertain which in vitro biofilm models will be most relevant to understanding detachment processes responsible for clinical cases of biomaterial centered infections. We propose that the biofilm model in our study will be useful for charactering aspects of early detachment events that may occur in catheters carrying a relatively rich medium such as vascular catheters delivering total parenteral nutrition.

## Methods

### Strains and media

*C. albicans *strain SC5314 was used for microarray analysis. Other strains used in this study are listed in Table 5. Stocks were stored in 10% glycerol at -80°C. A 1:1 dilution of standard YPD (0.5% bacto yeast extract, 1% bacto peptone, 1% glucose) was used for culturing both biofilms and planktonic (broth) cultures. This was supplemented with 1 mM L-arginine, 1 mM L-histidine and 0.5 mM uridine for culturing prototrophs. YPD was chosen for this study so comparisons with two other array studies could be made [36,37]. The carbon loading via glucose (55 mM) is similar to that used in other studies of *C. albicans *biofilm formation on biomaterials [72-74]

**Table 5:** *Candida albicans *strains used in the study

Strain	Strain	Genotype
AS1 (*ACT1-ALS3*)^1^	*ura3Δ::λimm434arg4::hisGhis1::hisG:: [pCIp-Act1] RPS1/RPS1:: [pCIp-Act1-ALS3]* *ura3Δ::λimm434 arg4::hisG his1::hisG*	[This study]
AS5 (*ACT1-ALS1*)	*ura3Δ::λimm434arg4::hisGhis1::hisG:: [pCIp-Act1] RPS1/RPS1:: [pCIp-Act1-ALS1]* *ura3Δ::λimm434 arg4::hisG his1::hisG*	[This study]
AS6 (*pga13/pga13*)	*ura3Δ::λimm434arg4::hisGhis1::hisGpga13Δ::URA3* *ura3Δ::λimm434 arg4::hisG his1::hisG pga13Δ::HIS1*	[This study]
AS9 (*psa2/psa2*)	*ura3Δ::λimm434arg4::hisGhis1::hisGpsa2Δ::ARG4* *ura3Δ::λimm434 arg4::hisG his1::hisG psa2Δ::HIS1*	This study]
AS10 (*ams1/ams1*)	*ura3Δ::λimm434his1::hisGarg4::hisGams1Δ::ARG4* *ura3Δ::λimm434 his1::hisG arg4::hisG ams1Δ::HIS1*	[This study]
BJ3a1a (*ywp1/ywp1*)	*ura3Δ::λimm434arg4::hisGywp1Δ::GFP-HIS1* *ura3Δ::λimm434 arg4::hisG ywp1Δ::GFP-HIS1*	[16]
BWP17	*ura3Δ::λimm434arg4::hisGhis1::hisG* *ura3Δ::λimm434 arg4::hisG his1::hisG*	[69]
CA14	*ura3Δ::λimm434* *ura3Δ::λimm434*	[70]
CAYF178U (*als3/als3*)	*ura3Δ::λimm434::URA3-IRO1als3::ARG4arg4::hisGhis1::hisG* *ura3Δ::λimm434 als3::HIS1 arg4::hisG his1::hisG*	[11]
CJN702 (*bcr1/bcr1*)	*ura3Δ::λimm434arg4::hisGhis1::hisG::pHIS1bcr1::ARG4* *ura3Δ::λimm434 arg4::hisG his1::hisG bcr1::URA3*	[11]
CJN459 (*bcr1/bcr1*)	*ura3Δ::λimm434arg4::hisGhis1::hisGbcr1::Tn7-UAU1* *ura3Δ::λimm434 arg4::hisG his1::hisG bcr1::Tn7-URA3*	[11]
CM-1613C (*mkc1/mkc1*)	*ura3Δ*::*λimm*434*mkc1Δ*::*hisG* *ura3Δ*::*λimm*434 *mkc1Δ*::*hisG*	[71]
DAY 286	*ura3Δ::λimm434ARG4:URA3::arg4::hisGhis1::hisG* *ura3Δ::λimm434 arg4::hisG his1::hisG*	[11]
FJS24 (*hwp1/hwp1*)	*ura3Δ::λimm434arg4::hisGhis1::hisGhwp1::Tn7-UAU1* *ura3Δ::λimm434 arg4::hisG his1::hisG hwp1::Tn7-URA3*	[unpublished^2^]
JC0188 (*aqy/aqy*)	*ura3Δ::λimm434aqyΔ::hisG-URA3-hisG* *ura3Δ::λimm434 aqyΔ::hisG*	[55]

^1 ^Simple genotype description for the mutants used in the study.

^2^Constructed by Frank J Smith

### Plasmid and C. albicans strain construction

Plasmid pFA-HIS1 was used as the template to prepare the HIS1-PCR cassette, and plasmid pFA-URA3 was used to prepare the *URA3*-PCR cassette as previously described [75]. They both have a common DNA backbone, allowing the same oligonucleotide pair to be used for the amplification of the two PCR cassettes. The oligonucleotides used in this work are listed and described (see Additional file 3). *AMS1*, *PSA2*, *PGA13 *and *CWH8 *genes were deleted in two steps. In the first step, one allele was replaced by homologous recombination with a PCR cassette containing the *HIS1 *gene. The BWP17 strain was transformed with the PCR fragments, and the cells were plated onto YPD plates minus histidine. Genomic DNA of the positive colonies was analyzed by PCR for the proper integration site of the cassette. The second allele disruption was accomplished as for the first allele using the *URA3*-PCR cassette and the cells were plated onto YPD plates minus uridine. The colonies were again analyzed by PCR to identify the proper integration site. The complete deletion of candidate genes was confirmed by PCR using primers internal to the recombination sites amplifying ORF regions. Growth rate of mutant strains in liquid medium was similar to the wild type strain.

### Biofilm cultures

Biofilms were cultured in a tubular reactor similar to that used in a previous study [29] (Figure 1). Biofilms were cultured in a 50 cm length of silicone elastomer tubing (Cole-Parmer, cat#96410-15) (5 mm ID, 10 mm OD), or, for one experiment, USP class VI medical grade silicone elastomer tubing of the same dimensions (P.E.P-plastics). Medium was pumped from a 2 L Erlemeyer flask using a peristaltic pump which was placed upstream of a flow break followed by the biofilm reactor. After sterilization by autoclaving the entire setup was placed in an incubator at 37°C. The inoculum was prepared as follows. 10 ml of broth was inoculated with a single colony from a YPD agar plate. Cultures were incubated on a shaker at 280 rpm and 30°C to an OD_600 _of 0.4–0.5. This was used to inoculate a fresh 10 ml broth culture at 0.05 OD_600 _which was grown overnight under the same conditions. From this culture 20 ml of cells at 10^8 ^cells/ml in phosphate buffered saline (PBS) (0.1 M, pH 7.0) was prepared. The tubular reactor was clamped downstream of the air trap and, using a 20 ml syringe, the reactor was filled by drawing the cell suspension into the tubing from the effluent end. The inoculated reactor was incubated for 1 h at 37°C before starting the medium flow at 1 ml/min.

### Planktonic cultures

Batch cultures were grown at 37°C on a shaker at 280 rpm. Preparation of the inoculum for planktonic cultures was the same as for biofilm cultures. The medium volume of batch cultures grown for different periods of time was adjusted so that the cells would be exposed to the same volume of medium as the biofilm for each time point. Accordingly, batch cultures were all inoculated with 1 × 10^8 ^cells and cultured in final volumes of 30, 60, 90, 120 and 180 ml for the 30, 60, 90, 120 and 180 min time points, respectively. For 90, 120 and 180 min time points the initial medium volume was 60 ml, and 30 ml aliquots of medium were added at appropriate times.

### Biofilm sectioning

Biofilms were sectioned using two methods. For embedding in Spurr's resin [76] biofilm samples were fixed in situ at 4°C in 3% gluteraldehyde in PBS. The fixed samples were washed at room temperature for 10 min in 20, 50 and 100% ethanol solutions successively. Samples were incubated in a series of Spurr's: 1:2 Spurr's: propylene oxide (overnight at 4°C); 1:1 Spurr's: propylene oxide (8–10 h at room temperature), 2:1 Spurr's: propylene oxide (overnight at 4°C) and full strength Spurr's (6–8 h at room temperature). The Spurr's solution of the last incubation was replaced by a fresh one and samples were baked for 10–12 h in an oven at 70°C. After cooling to room temperature, the silicone tube was removed from each sample and the hardened Spurr's column containing the biofilm was sectioned using a Reichert OM-U2 ultramicrotome. Sections were mounted on slides and imaged using a Nikon Eclipse E600 in epi-fluorescence mode.

Samples for cryosectioning were prepared by excising a section of the silicone elastomer tube used to grow the biofilm with a fresh razor blade without disturbing the biofilm. Excess medium in the tube was carefully removed using a 10 ml syringe and needle. The tubing was cut lengthwise and the upper half was removed. The biofilm on the lower half was covered carefully with a layer of OCT (optimum cutting temperature formulation, Tissue-Tek) and placed on dry ice until completely frozen. The tubing was carefully peeled away from the frozen biofilm by warming up the tube part briefly between fingers. The frozen biofilm sample was dipped vertically into the center of a cryosectioning cup filled with fresh OCT which was placed on dry ice until it was completely frozen. Frozen samples were sectioned at -19°C using a Leica CM1850 cryostat. The 5 μm thick cryosections were mounted on Superfrost/Plus microscope slides (Fisher Scientific), washed gently with distilled water to remove the excess OCT and dried at room temperature. Cryosections were imaged using a Nikon Eclipse E800 microscope interfaced to a Metaview 2.0 image acquisition system (Molecular Devices). Unstained sections were viewed in transmission using DIC optics. Sections stained with calcofluor (Fungi-Fluor™ stain, Polysciences, Inc) were viewed in epi-fluorescence mode.

### Antibody labeling of (1,3) β glucan in biofilm cryosections

The protocol for staining biofilm cryosections for (1,3) β glucan was a modification of a published protocol [77]. The primary monoclonal antibody (mAb) was from Biosupplies Australia (produced in mice). The secondary anti-mouse antibody, conjugated to Alexa Fluor 488, was from Invitrogen (produced in rabbits). We used planktonic cells grown at 30°C and adhered to slides used for cryosectioning (Superfrost/Plus microscope slides, Fisher Scientific) as positive and negative controls. The negative control was omission of the primary antibody. In this case no fluorescence was detected under exposure conditions in which there was relatively bright fluorescence originating from cells exposed to the primary antibody. In addition, fluorescence was in every case associated with cells as confirmed by comparing images acquired using epi-fluorescence and transmission modes (data not shown). OCT was rinsed from the biofilm cryosections before antibody staining using Tween Tris Buffered Saline (TTBS), pH 7.6. This was followed by exposure to TTBS with 1% BSA (15 min), exposure to the primary mAb at 4 ug per ml in TTBS (1 h), three washes with TTBS (5 min each), exposure to the secondary Ab at a 1:100 dilution in TTBS (30 min) and a wash with TTBS 3 times (5 min each).

### Digital camera images and movies

Digital camera images were acquired using an Olympus SP-350 8 Megapixels digital camera at the highest resolution mode. Digital movies were recoded using a QX5 Computer Microscope (Digital Blue Inc.).

### Cell counts and hyphal length

Both biofilms and planktonic cultures were exposed to 20 mg/ml pronase in Tris buffer (10 mM Tris/HCl, pH 8.0, 2 mM EDTA) for 60 min to disperse cell aggregates according to a previously published protocol. [78] (Cell aggregates could not be dispersed sufficiently for either counting or hyphal length measurement by vortexing alone). Cells were counted in a hemacytometer. Hyphal length was measured from images acquired of dispersed cells using the Nikon/Metaview system described above.

### Field emission scanning electron microscopy (FESEM)

The tubing was first drained as described in the following section. A tube section was excised and cut lengthwise into two pieces. The bottom part, where the cells settle and form the biofilm, was immersed overnight in fixing buffer (1% paraformaldehyde, 2.5% gluteraldehyde in 0.1 M sodium cacodylate buffer, pH 7.2–7.4). The fixed samples were rinsed twice for 10 min in 0.1 M sodium cacodylate buffer and dehydrated twice for 5 min in 50%, 70%, 90% and 100% ethanol solutions. Samples were dried at room temperature. Samples were coated with a thin film of iridium, 15 s at 20 mA, in a Emitech sputter coater. Cells were viewed with a Supra 55VP FESEM (Zeiss) using the Inlens detector at 1 kV and 3 mm working distance.

### RNA preparation

Biofilm samples were collected by first clamping and then removing the colonized section of the tubing. The liquid column was drained into a 50 ml polypropylene tube placed in an ice bath by moving the tubing to a vertical position and releasing the clamps. For 1 h biofilms the more firmly attached biofilm was then removed by rolling the tubing between the hands followed by flushing the tube with 25 ml of ice-cold RNase-free water using a 50 ml syringe to achieve the highest pressure possible. This procedure was accomplished in less than 3 min for each experiment. Cells from batch cultures were collected by pouring the contents of the culture flask into 50 ml polypropylene tubes in an ice bath. Cells from biofilm or batch cultures were centrifuged at 4°C in 10–20 ml aliquots at 2500 × g for 3 min, washed with ice-cold RNase-free H_2_O and immediately flash-frozen in liquid N_2 _and stored at -80°C until use.

To release the RNA from cells, samples stored at -80°C were placed on ice and RNeasy buffer RLT was added to pellets at a ratio of 10:1 [vol/vol] buffer/pellet. The pellet was allowed to thaw in the buffer while vortexing briefly at high speed. The resuspended pellet was placed back on ice and divided into 1 ml aliquots in 2 ml screw cap microcentrifuge tubes containing 0.6 ml of 3 mm diameter acid-washed glass beads. Samples were homogenized 5 times, 1 min each, at 4200 RPM using the Mini-Beadbeater mill (Biospec Products Inc., Bartlesvile, OK, USA). Samples were placed on ice for 1 min after each homogenization step. After the homogenization the Qiagen RNeasy protocol was followed as recommended. Total RNA samples were eluted in RNase free H_2_O, flash-frozen in liquid N_2_, lyophilized and stored at -80°C until used for the different analyses.

### Microarray experiments: cDNA labeling, hybridizations and data analysis

Four independent biological replicates were performed for each hybridization comparison. Labeling of the four biological replicate was performed using a dye-swap strategy that resulted in 2 experiments with Cy3/Cy5 and two experiments Cy5/Cy3 ratios. RNA quality and integrity were assessed using an Agilent 2100 Bioanalyzer. cDNA labeling and microarray production were performed as previously described by Nantel et al. [79]. Briefly, 20 μg of total RNA was reverse transcribed using oligo(dT)_21 _in the presence of Cy3 or Cy5-dCTP (Invitrogen) and Superscript III reverse transcriptase (Invitrogen). Thereafter, template RNA was degraded by adding 2.5 units RNase H (USB) and 1 μg RNase A (Pharmacia) followed by incubation for 15 min at 37°C. The labeled cDNAs were purified with QIAquick PCR Purification Kit (Qiagen). Prior to hybridization Cy3/Cy5-labeled cDNA was quantified using a NanoDrop ND-1000 UV-VIS spectrophotometer (NanoDrop) to confirm dye incorporation. Pre-hybridization and hybridization solutions consisted of DIG Easy Hyb solution (Roche Diagnostics, Mannheim, Germany) with 0.45% salmon sperm DNA and 0.45% yeast tRNA. Slides were washed once in 1.0% SSC, 0.2% SDS at 42°C for 10 min, twice in 0.1% SSC, 0.2% SDS at 42°C for 10 min, once in 0.1% SCC at 24°C for 5 min, followed by four rinses in 0.1% SSC. Chips were air dried before being scanned using a ScanArray Lite microarray scanner (Perkin Elmer). QuantArray was used to quantify fluorescence intensities. Data handling, analysis and normalization were carried out using Genespring GX v.7.3 (Agilent Technologies, CA). Genes with statistically significant changes in transcript abundance in each experiment were identified using a 1.5-cutoff and Welch t-test with a False Discovery Rate (FDR) less than 5%. Gene annotations were from http://www.candidagenome.org or http://www.yeastgenome.org. The latter database was accessed using the DAVID search program [80].

### Expression analysis by real-time quantitative PCR

cDNA was synthesized from 5 μg of total RNA using the reverse-transcription system (50 mm Tris-HCl, 75 mm KCl, 10 mm dithiothreitol, 3 mm MgCl_2_, 400 nm oligo(dT)_15_, 1 μm random hexamers, 0.5 mm dNTP, 200 units Superscript II reverse transcriptase; Invitrogen). The total volume was adjusted to 20 μL and the mixture was then incubated for 60 min at 42°C. Aliquots of the resulting first-strand cDNA were used for real-time PCR amplification experiments. Real-time PCR was performed using the Corbett Rotor-Gene RG-3000A (Corbett Research, Sydney, Australia) with the SYBR Green PCR master mix (Qiagen) according to the manufacturer's instructions. After 10 min denaturation at 95°C, the reactions were cycled 40 times at 95°C for 15 s, 56°C for 15 s and 72°C for 30s. To verify that only the specific product was amplified, a melting point analysis was performed after the last cycle by cooling samples to 55°C and then increasing the temperature to 95°C at 0.2°C per second. A single product at a specific melting temperature was found for each target. All samples were tested in triplicate and the mean was determined for further calculations. Each run included a no template control to test for assay reagent contamination. To evaluate the gene expression level, the results were normalized using Ct values obtained from Actin (*Act1*, orf19.5007). The relative quantification analysis was performed using the comparative Ct method as described by Guillemette et al. [81].

### Estimation of shear stress

Shear stress (τ) is defined as:

(1) τ = μ(dv/dy)

where μ is the absolute (dynamic) viscosity (approximately 10^-2 ^dynes sec cm^-2^). For a cylindrical geometry the slope of the velocity profile at the tube wall (dv/dy) is related to the maximum velocity (V_max_) by:

(2) dv/dy = 2(V_max_/r)

where r is the radius of the tubing and:

(3) V_max _= 2 V

where V is the mean flow velocity across the velocity profile (the volumetric flow divided by the cross sectional area of the interior of the tubing). The shear stress applied in draining the tubing was estimated from the average V determined from the time required for the medium plug to reach the end of the tubing (0.5s).

## Authors' contributions

AS performed microarray analysis, constructed mutant strains, did PCR analysis and contributed to analysis of array data. TA cultured and characterized biofilms, and collected and purified RNA for array analysis. KM contributed to analysis of array data, particularly to K means analysis. SB performed TEM analysis. AN was primarily responsible for the design and analysis of the microarray experiments and especially the comparison with other data sets. PAS performed SEM and microscopy, contributed to array analysis and was primarily responsible for biofilm experimental design.

## Supplementary Material

Additional file 1 **Biofilm Time Course Array Dataset.** Complete list of differentially regulated genes Click here for file

Additional file 2 **Biofilm versus Batch Time Array Dataset.** Complete list of differentially regulated genes Click here for file

Additional file 3 **Primers used in this study.** Primer sequences used to construct the mutant strains Click here for file
