# Supplementary material for: A Candida albicans early stage biofilm detachment event in rich medium
Source: BMC Microbiol. 2009 Feb 2;9:25. doi: 10.1186/1471-2180-9-25 (PMC2647545; doi:10.1186/1471-2180-9-25)
Supplement: Additional file 3 — Primers used in this study. Primer sequences used to construct the mutant strains [file 1471-2180-9-25-S3.doc]

Table 2. Primers used in the study

| **Primer** | **Primer sequence ^1^** | **Description** | **Purpose** |
| --- | --- | --- | --- |
| AMS1 F1 | Ctttcaaattgttcttttcaactttttattactttatttcttcttctcccaatattgattttatttactttcctaactagctagtactatttctatcat**ggaagcttcgtacgctgcaggtc** | 5' AMS1 PCR cassette (122 nt) | Disruption of *AMS1* |
| AMS1 R1 | Gtcttttttatttatttatttatttattcattgatttatttatataactatctaaaaactttgtcctttttttttttattttttattttattttatttt**atctgatatcatcgatgaattcgag** | 3' AMS1 PCR cassette (98 nt) | Disruption of *AMS1* |
| CWH8 F1 | Catctatcaaaaatttgcagcttttagttatttgatattatttgttatttgttatttgttatttgttgtttgttattcatttgcttttcaat**ggaagcttcgtacgctgcaggtc** | 5' CWH8 PCR cassette (92 nt) | Disruption of *CWH8* |
| CWH8 R1 | Caacaacaaaatggaaatcaatcaatcaatcaatcaactaaaatattctattcaacttttttttttatctattctccaagtgatatatctttttt**atctgatatcatcgatgaattcgag** | 3' CWH8 PCR cassette (94 nt) | Disruption of *CWH8* |
| PSA2 F1 | Gtattgtagattttatatattgtttgtctttctttttcaactttgttttatcaaagagtttttcatctacatacgttgaaccatattagtta**tggaagcttcgtacgctgcaggtc** | 5' PSA2 PCR cassette (93 nt) | Disruption of *Psa2* |
| PSA2 R1 | Caaaatgatagataggttataaggttatgtttattgtatttttataggaatttagaatttgataaggttatttttaaggctatttttcttcatctc**atctgatatcatcgatgaattcgag** | 3' PSA2 PCR cassette (93 nt) | Disruption of *Psa2* |
| PGA13 F1 | GTGATAGGAAAAAAAAAGTATAAATAGATCCATGGTTTTCCAATATTTTGCATTCACTGTAGACTAGGTTTATAACACACCATCATAATCATTCGCTATG**ggaagcttcgtacgctgcaggtc** | 5' PGA13 PCR cassette (100 nt) | Disruption of *PGA13* |
| PGA13 R1 | TAAGAAGTCAGCAACAGTTTAAAAATGAAAGCAATAATGAAATTGTCTTTGATGTCGTTGTTGAGGCATAACTCTTAGATTGATAATGCCAACAATATCt**atctgatatcatcgatgaattcgag** | 3' PGA13 PCR cassette (99 nt) | Disruption of *PGA13* |
| AMS1 ExF1 | Gtaattgtgttatggtgtgctgtc | AMS1, forward-external | Validation of  5’ cassette insertion |
| AMS1 ExR1 | Aattcgtagtaaattaatggaccta | AMS1, reverse-external | Validation of  3’ cassette insertion |
| AMS1 InF1 | Atccagccagtgtttccaag | AMS1, forward-internal | Detection of *AMS1 alleles* |
| AMS1 InR1 | Catgaattgctccaccatatttaga | AMS1, reverse- internal | Detection of *AMS1 alleles* |
| CWH8 ExF1 | Tacccagttatcccaatatttctggtc | CWH8, forward-external | Validation of  5’ cassette insertion |
| CWH8 ExR1 | Gtggatcaaagaaaaacgatgatg | CWH8, reverse-external | Validation of  3’ cassette insertion |
| CWH8 InF1 | Ccaaaatttgtgttcaattttcat | CWH8, forward-internal | Detection of *CWH8 alleles* |
| CWH8 InR1 | Ttggccaatttaaaatccattc | CWH8, reverse- internal | Detection of *CWH8 alleles* |
| PSA2 ExF1 | Cattatactatattttaaggacaaatga | PSA2, forward-external | Validation of  5’ cassette insertion |
| PSSA ExR1 | Agatatactcaagtatgacgaacca | PSA2, reverse-external | Validation of  3’ cassette insertion |
| PSA2 InF1 | Tgcgggtggattataccatt | PSA2, forward-internal | Detection of *PSA2 alleles* |
| PSA2 InR1 | Ctgttgtcgttgtcgttgct | PSA2, reverse- internal | Detection of *PSA2 alleles* |
| PGA13 ExF1 | AGTGAAGCCATTGACCAACC | PGA13, forward-external | Validation of  5’ cassette insertion |
| PGA13 ExR1 | GCTGGAAGGTCCCCTCTTAT | PGA13, reverse-external | Validation of  3’ cassette insertion |
| PGA13 IntF1 | Gaagcgttgtcaccactcaagtcacc | PGA13, reverse- internal | Detection of *PGA13 alleles* |
| PGA13 IntR1 | Tggcttttctgaagctggcac | PGA13, forward-internal | Detection of *PGA13 alleles* |
| H1 | TTTAGTCAATCATTTACCAGACCG | HIS1, forward | Validation of  3’ cassette insertion |
| H2 | TCTATGGCCTTTAACCCAGCTG | HIS1, reverse | Validation of  5’ cassette insertion |
| U1 | TTGAAGGATTAAAACAGGGAGC | URA3, forward | Validation of  3’ cassette insertion |
| U2 | ATACCTTTTACCTTCAATATCTGG | URA3, reverse | Validation of  5’ cassette insertion |
| Dpm2 F1 | Tggtaatttgatggcataatc | DPM2, forward | Real time quantitative PCR validation |
| Dpm2 R1 | Ttggatttggaatgcttggt | DPM2, reverse | Real time quantitative PCR validation |
| Dpm3 F1 | gtaacaattccccaacctaacg | DPM3, forward | Real time quantitative PCR validation |
| Dpm3 R1 | Caggtgtgattccaactcca | DPM3, reverse | Real time quantitative PCR validation |
| Wbp1 F1 | Ttacttggttcgtcggctt | WBP1, forward | Real time quantitative PCR validation |
| Wbp1 R1 | aacatggacctttggtgagc | WBP1, reverse | Real time quantitative PCR validation |
| Erv29 F1 | tggccaccaaaaatgctcta | ERV29, forward | Real time quantitative PCR validation |
| Erv29 R1 | tggtaatcaacctccacatca | ERV29, reverse | Real time quantitative PCR validation |
| Sec22 F1 | Atccaaagaatcgccccta | SEC22 forward | Real time quantitative PCR validation |
| Sec22 R1 | cgaagctttaagtaataaagccaga | SEC22, reverse | Real time quantitative PCR validation |
| Emp24 F1 | gttggcagcaacaacaaaga | EMP24, forward | Real time quantitative PCR validation |
| Emp24 R1 | atgtcgatgtgaacgatcca | EMP24, reverse | Real time quantitative PCR validation |
| Chs7 F1 | Cacccaatgaccaatatgattcta | CHS7, forward | Real time quantitative PCR validation |
| Chs7 R1 | ttgcatggtttgctgtcaat | CHS7, reverse | Real time quantitative PCR validation |
| Yop1 F1 | acaccttcagcggtttcttg | YOP1, forward | Real time quantitative PCR validation |
| Yop1 R1 | tcaaacaccatcttatactatgtccca | YOP1, reverse | Real time quantitative PCR validation |
| Sss1 F1 | taacggcatagccgacaac | SSS1, forward | Real time quantitative PCR validation |
| Sss1 R1 | tggctgctgaaggattagaaa | SSS1, reverse | Real time quantitative PCR validation |

^1^Primer sequences are given in the 5' to 3' direction. Sequences in bold are segments of the primers that anneal to plasmids pFA-His1 and pFA-URA3 for amplification of the disruption cassettes used in constructing the mutants [34].
